# Whole-Genome Sequencing of *Flammulina filiformis* and Multi-Omics Analysis in Response to Low Temperature

**DOI:** 10.3390/jof11030229

**Published:** 2025-03-17

**Authors:** Xinmin Liang, Jing Han, Yuqin Cui, Xueqin Shu, Mengting Lei, Bo Wang, Dinghong Jia, Weihong Peng, Xiaolan He, Xun Liu

**Affiliations:** 1 Sichuan Institute of Edible Fungi, Sichuan Academy of Agricultural Sciences, Chengdu 610066, Chinaxiaolanhe1121@aliyun.com (X.H.); 2 Key Laboratory of Coarse Cereal Processing, Ministry of Agriculture and Rural Affairs, College of Food and Biological Engineering, Chengdu University, Chengdu 610106, China; 3 College of Resources, Sichuan Agricultural University, Chengdu 611130, China

**Keywords:** *Flammulina filiformis*, whole-genome sequencing, transcriptome, metabolome, cold adaptation

## Abstract

The growth of *Flammulina filiformis* is strongly dependent on low-temperature cues for the initiation of primordia formation. To obtain a comprehensive understanding of the molecular mechanisms that govern the mycelial response to cold stress, de novo genome sequencing of the *F. filiformis* monokaryon and multi-omics data (transcriptome and metabolome) analyses of the mycelia, primordia, and fruiting bodies were conducted in the present study. Genome sequencing based on PacBio HiFi and Hi-C resulted in a 36.3 Mb genome sequence that mapped to 12 chromosomes, comprising 11,886 protein-coding genes. A total of 25 cold-responsive (COR) genes and 520 cold-adapted enzymes were identified in the genome. Multi-omics analyses showed that the pathways related to carbohydrate metabolism in the mycelia under low temperature (10 °C) were significantly enriched. Further examination of the expression profiles of carbohydrate-active enzymes (CAZymes) involved in carbohydrate metabolism revealed that out of 515 CAZyme genes in *F. filiformis*, 58 were specifically upregulated in mycelia under low-temperature conditions. By contrast, the expression levels of these genes in primordia and fruiting bodies reverted to those prior to low-temperature exposure. These indicate that CAZyme genes are important for the low-temperature adaptation of *F. filiformis*. This research contributes to the targeted breeding of *F. filiformis*.

## 1. Introduction

*Flammulina filiformis*, commonly referred to as the winter mushroom, was identified as *F. velutipes* in the previous literature in China [1]. It is an important edible mushroom extensively cultivated throughout East Asia, particularly in China, Japan, Vietnam, and Korea [2]. In 2022, China’s total production of *F. filiformis* reached 2.025 million tons, reflecting its widespread cultivation and commercial importance [3]. The growth and development of *F. filiformis* are highly sensitive to environmental factors, necessitating the precise regulation of temperature. Previous research has demonstrated that exposure to cold stress can cause the aerial mycelia of *F. filiformis* to become flattened and secrete yellow physiological water [4,5]. Additionally, temperature variations can influence the sugar content of the fruiting bodies [6]. Interestingly, the mycelium of *F. filiformis* initially undergoes vegetative growth in the culture medium, subsequently transitioning to reproductive growth in response to low-temperature stimuli. Cooling is essential for promoting the differentiation of primordia. In its natural habitat, *F. filiformis* typically fruits from late autumn to early spring, a period characterized by low temperature [7]. This observation underscores the critical role of low temperature in the formation of primordia and fruiting bodies. Research has demonstrated that temperatures below 15 °C are generally required to induce primordia formation, whereas fruiting-body differentiation necessitates even lower temperatures, ranging from 4 °C to 6 °C [8]. Despite the acknowledged significance of low temperature in the life cycle of *F. filiformis*, the molecular mechanisms underlying its adaptation to such conditions remain insufficiently explored. In plants, the ICE–CBF–COR (CBF expression inducer–C-repeat binding factor–cold regulation) pathway is one of the widely studied low-temperature response mechanisms [9]. When plants are exposed to low temperature, the ICE transcription factors upstream of CBF increase their expression, thereby promoting the transcription of CBF genes, which, in turn, enhances the expression of downstream COR genes [10]. Some COR genes encode antifreeze proteins or enzymes involved in the biosynthesis of osmotic adjustment substances or the scavenging of reactive oxygen species, thereby improving the cold resistance of plants [9,11,12]. Previous reports indicate that fungi produce cold-adapted enzymes under low temperatures, including hydrolases (such as lipases, proteases, cellulases, etc.) and oxidoreductases (such as laccases and superoxide dismutases), which help them survive in cold environments [13].

Genomic data are anticipated to have substantial applications in the genetic engineering and breeding of edible fungi. This data, including species such as *Pleurotus giganteus*, *Oudemansiella raphanipes*, pink *Auricularia cormea*, *Stropharia rugosoannulata*, *Morchella eohespera*, etc., provide valuable resources for precise genetic improvement and the identification and mining of functional genes [14,15,16,17,18]. The genome sequencing assembly of *F. filiformis* was first reported at the chromosomal level [19], showing that its genome was assembled into 11 chromosomes. However, subsequent assemblies of other *F. filiformis* strains have revealed 12 chromosomes in the NCBI database. Currently, there is no consensus on the exact number of chromosomes in *F. filiformis*. Although the whole-genome sequencing of *F. filiformis* has been completed, its gene annotation data are not yet fully open access, which somewhat limits its potential for functional gene research and genetic improvement. Meanwhile, the application of genomic information will facilitate the development of gene editing technologies, enabling researchers to directly improve the traits of edible fungi, thereby enhancing their yield, quality, disease resistance, and environmental adaptability, among other characteristics. For instance, a previous study identified the key genes for the synthesis of γ-aminobutyric acid (GABA) in *F. filiformis* through genome mining and verified them through overexpression, laying the foundation for the directional breeding strategy for high-yield GABA [20].

Multi-omics technologies offer powerful tools for studying the biological characteristics of edible fungi. By integrating various data such as genomics, transcriptomics, proteomics, and metabolomics, it is feasible to deeply analyze the adaptation mechanisms of organisms in different biological environments or reveal the molecular formation mechanisms in specific biological changes. For instance, in the plant species *Salvia rosmarinus*, comprehensive genomic, transcriptomic, and metabolomic analyses have demonstrated that abiotic stress treatments lead to the upregulation of 36 genes involved in the biosynthetic pathways of carnosic acid, rosmarinic acid, and flavonoids. This upregulation underscores the significant role these antioxidant compounds play in mitigating abiotic stress by regulating reactive oxygen species (ROS) homeostasis [21]. In the case of edible fungi, a thorough examination of the genome, transcriptome, and proteome has preliminarily identified oxidation as the primary cause of postharvest browning in *F. filiformis*. This analysis has also uncovered the regulatory genes and metabolic pathways implicated in oxidative browning [22]. Previous research utilizing de novo transcriptome sequencing of mycelia at 25 °C and primordia at 10 °C has preliminarily identified candidate genes, such as histidine kinase and MAPK kinase, that may be involved in the low-temperature-induced fruiting of *F. filiformis* [23].

In the present work, to elucidate the adaptation mechanisms of *F. filiformis* mycelium in low-temperature environments, the whole genome of *F. filiformis* was sequenced and assembled, and the composition and function of the genome were analyzed. Multi-omics analysis was employed to screen out the pathways and genes related to the low-temperature response, and the expression profile of the CAZyme gene was identified. The results provide valuable insights for subsequent research on the gene function and genetic improvement of *F. filiformis*.

## 2. Method and Materials

### 2.1. Strains and Cultural Conditions

The dikaryotic *F. filiformis* yellow strain HNY6, a wild *F. filiformis* collected from Henan Province, China, was acquired through tissue isolation, and the monokaryotic strain HNY6m1 was derived from spore isolation of HNY6 strain. The fruiting test of HNY6 was conducted according to the method described in the previous study [24], with some modifications. The cultivation substrate was composed of 69% cottonseed hulls, 20% rice bran, 10% wheat bran, and 1% CaCO_3_, with a water content of 65%, and was sterilized by vertical high-pressure steam sterilizer (LDZM-80L-I, Shenan, China) at 121 °C for 150 min before use [25]. The strain was initially cultivated in potato dextrose agar (PDA) medium and then inoculated into plastic bottles filled with the cultivation substrate, which were placed under dark conditions at 25 °C until the mycelium filled the bottles. Subsequently, the surface strain blocks and mycelium skins were removed, and they were transferred to 10 °C for the primordia formation. After primordia formation, the cultures were transferred to 4 °C for fruiting-body development. The mycelia incubated at 25 °C (M), and the mycelia (ML) cultivated at 10 °C were collected for transcriptomic and metabolomic analyses. Additionally, transcriptomic analysis was conducted on the primordia (P) and the upper, middle, and lower parts of the fruiting bodies. Metabolomic and transcriptomic sequencing were performed at the Beijing Novogene Bioinformatics Technology Co., Ltd (Beijing, China).

### 2.2. Genome Sequencing and Assembly

The *F. filiformis* HNY6m1 strain was inoculated on PDA plates that were covered with cellophane and cultured at 25 °C for 7 d; 3 g of mycelia was scraped for genome DNA extraction. Genome DNA was extracted with the SDS method [26]. The harvested DNA was detected by agarose gel electrophoresis and quantified by Qubit fluorometer (v2.0, Thermo Scientific, Waltham, MA, USA). For next-generation sequencing, sequencing libraries were generated using NEBNext^®^ Ultra™ DNA Library Prep Kit for Illumina (NEB, Ipswich, MA, USA) following the manufacturer’s protocol. Briefly, the DNA sample was fragmented by sonication to a size of 350 bp using an ultrasonic homogenizer (Covaris, Woburn, MA, USA), and then DNA fragments were end-polished, A-tailed, and ligated with the full-length adaptor for Illumina sequencing with further PCR amplification. At last, PCR products were purified (AMPure XP system, Beckman Coulter, Brea, CA, USA), and libraries were analyzed for size distribution by Agilent2100 Bioanalyzer (Agilent, Santa Clara, CA, USA). The whole genome of *F. filiformis* was sequenced using Illumina NovaSeq PE150 platform for genome survey and assessment. For third-generation sequencing, libraries were constructed using SMRT bell TM Template kit (version 2.0) (Pacific Biosciences, Menlo Park, CA, USA). The genomes were sequenced using the PacBio Sequel II platform (Pacific Biosciences) to produce HiFi reads. Falcon software (version 0.3.0) (https://github.com/PacificBiosciences/FALCON/) (accessed on 21 September 2024) [27] was utilized for genome assembly based on HiFi reads, resulting in a preliminary assembly. Subsequently, three rounds of error correction were performed using Racon software (version: 1.4.13) with third-generation sequencing reads, followed by three rounds of error correction using Pilon software (version: 1.22) with second-generation reads to obtain the final assembled genome.

The Hi-C library was constructed and sequenced on an Illumina NovaSeq PE150 platform by Beijing Novogene Bioinformatics Technology Co., Ltd. Based on the HIC sequencing reads, LACHESIS software (version 1.0) (http://shendurelab.github.io/LACHESIS/) (accessed on 21 September 2024) was utilized for genome sequence clustering, sorting, and orientation. Manual correction was performed using Juicebox (version 1.5.1.2) (https://github.com/aidenlab/Juicebox) (accessed on 21 September 2024), resulting in a genome assembly at nearly chromosome level. BUSCO (version 5.8.2) (Benchmarking Universal Single-Copy Orthologs: http://busco.ezlab.org/) (accessed on 21 September 2024) was used to assess the final genome assembly. The genome has been submitted to the NCBI under the accession number PRJNA1110060.

### 2.3. Gene Prediction and Annotation

The Augustus [28], GlimmerHMM [29], and SNAP programs [30] were employed for de novo gene structure prediction. Furthermore, RNA-seq data from *F. filiformis* mycelium and primordia were utilized to identify protein-coding genes and transcripts through TransDecoder (version 5.7.1) (https://github.com/TransDecoder/TransDecoder) (accessed on 21 September 2024) and PASA programs (version 2.0.2). Subsequently, the results predicted by multiple methods were integrated using EVM (version 1.1) and underwent a second-round validation with PASA [31]. Finally, gene models predicted by the aforementioned methods were corrected using RNA-seq data and then consolidated into a non-redundant and complete gene set.

Functional annotations of genes were predicted in assembled genome of *F. filiformis* according to the following databases: Non-Redundant (NR), Pfam, SwissProt, Gene Ontology (GO), and Kyoto Encyclopedia of Genes and Genomes (KEGG). Then, the predicted genes were classified based on their functions using the GO and KEGG databases for GO functional classification and KEGG functional classification, respectively.

### 2.4. Comparative Genomic Analysis

The 14 fungal species with complete genomes and annotation information published in the NCBI database were selected for comparative genomic analysis with *F. filiformis* in this study. Among the 14 kinds of fungi, there are 9 basidiomycetes (*Agaricus bisporus*, *Coprinopsis cinerea*, *Hypsizygus marmoreus*, *Laccaria bicolor*, *Volvariella volvacea*, *Lentinula edodes*, *Pleurotus ostreatus*, *Serpula lacrymans*, and *Hericium alpestre*) and 5 ascomycetes (*Aspergillus nidulans*, *Neurospora crassa*, *Morchella importuna*, *Morchella sextelata*, and *Saccharomyces cerevisiae*).

The gene families and single-copy orthologous genes of the genomes of the above-mentioned species were analyzed using the Orthofinder (v2.5.4) software. The proteins of the genomes were pairwise compared using the BLAST software (Version 2.2.26), and the unreliable comparison results were filtered. Then, the proteins were clustered according to the comparison similarity using Hcluster-sg (Version 0.2.0).

### 2.5. Identification of Cold-Responsive Genes and Cold-Adapted Enzymes

The current techniques for identifying key genes and studying gene functions in edible fungi are primarily based on approaches used in plant research. Therefore, in this study, we predicted the genes potentially involved in cold response in the *F. filiformis* genome from previous reports [32]. Cold-signal-responsive proteins were selected from *Arabidopsis thaliana* as references. According to the TAIR GO annotation (https://www.arabidopsis.org/) (accessed on 11 February 2025), 67 proteins labeled as “cold acclimation (CA)”, 35 proteins labeled as “cellular response to cold (CRC)”, and 386 proteins labeled as “response to cold (RC)” were screened out. Subsequently, BLASTP in Tbtools [33] software (version 2.142) was used for alignment analysis, and the search thresholds were set as follows: e-value ≤ 1 × 10^−30^ and similarity ≥ 50%. According to previous reports [13], the cold-adapted enzymes in the genome of *F. filiformis* were identified using the gene annotation information in the genome assembled in this study.

### 2.6. Transcriptome Analysis

According to the manufacturer’s instructions, total RNA was extracted using TRIzol reagent (Invitrogen, Carlsbad, CA, USA), followed by RNA purification using the RNeasy kit and RNase-free DNase. The purified RNA was subjected to high-throughput sequencing on the Illumina platform (Novogene, Beijing, China). Next, Fastq software (version 2.19) was used for quality control and data filtering to remove low-quality sequences and adapter contamination. Subsequently, the quality-controlled data were mapped to the genome sequence of *F. filiformis* using HISAT2 software (version 2.0.5). Finally, the number of genes in the samples was analyzed by FeatureCounts software (version 1.5.0-p3), and the expression levels of each gene were calculated using FPKM (fragments per kilobase of exon model per million mapped fragments). In addition, DESeq2 software (version 1.20.0) was used for differential expression gene (DEG) analysis, and the screening criteria were set as |log2 Fold Change| ≥ 1 or 2 and padj < 0.05 to identify genes that changed significantly between the two groups of samples. Functional annotation and pathway analysis of these DEGs were conducted using Gene Ontology (GO) and Kyoto Encyclopedia of Genes and Genomes (KEGG) databases, respectively.

### 2.7. Metabolome Analysis

The collected *F. filiformis* samples were ground into fine powder in liquid nitrogen, extracted with 80% methanol, vortexed, incubated on ice for 5 min, then centrifuged at 12, 000 rpm at 4 °C for 20 min using a centrifuge (LEGEND MICRO 21R, Thermo Scientific, Waltham, MA, USA). The supernatant was taken, and the methanol concentration was diluted to 53% with mass spectrometry-grade water. Then, metabolomic analysis was performed using an ultra-performance liquid chromatography coupled to tandem mass spectrometry (UPLC–MS/MS) system (Q Exactive™ HF, Vanquish UHPLC, Thermo Scientific, Waltham, MA, USA) and a Hypersil Gold C18 column (5 µm, 150 × 4.6 mm, Thermo Scientific, Waltham, MA, USA). The mobile phase A was 0.1% formic acid aqueous solution, and the mobile phase B was methanol. The gradient used was 2% B within 2 min, increased to 85% at 3 min and maintained for 7 min, increased to 100% at 10 min, maintained for 3 min, then decreased to 2% and maintained until 15 min. The flow rate was 0.2 mL/min, and the injection volume was 5 μL. The mass spectrometry condition parameters are as follows: electrospray ionization source, spray voltage of 3.5 kV, sheath gas flow rate of 35 psi, auxiliary gas flow rate of 10 L/min, ion transfer tube temperature of 320 °C, ion introduction radio frequency level of 60, auxiliary gas heater temperature of 350 °C, and mass scan range of *m*/*z* 100–1500. The identification of metabolites was carried out by comparing the *m*/*z* values and MS/MS spectra with the standard established internal database (imz Cloud, mz Vault and Masslist). The putative metabolite candidates were further screened based on the fragmentation score, and only those with a score ≥ 50 were used for compound identification. Metabolites with VIP > 1, FC > 1.5 or FC < 0.667, and padj < 0.05 were regarded as differential accumulated metabolites (DAMs). KEGG was used to annotate the biochemical pathways involved in these DAMs.

### 2.8. Identification and Expression Profile of CAZymes

According to the carbohydrate-active enzymes (CAZymes) database [34], the genes with putative CAZymes were annotated through BLASTP analysis. Based on the transcriptome data of *F. filiformis* mycelia under low-temperature treatment and room-temperature culture, the significantly differentially expressed CAZymes genes were screened out. The gene expression heatmap was drawn using the TBtools software [33] based on the FPKM values of these genes in different tissues, and the expression profiles of these genes in the upper, middle, and lower tissues of the fruiting body of *F. filiformis* were further analyzed based on the FPKM values of the genes.

## 3. Results

### 3.1. The Genome Assembly and Annotation of F. filiformis

**Figure 1:**
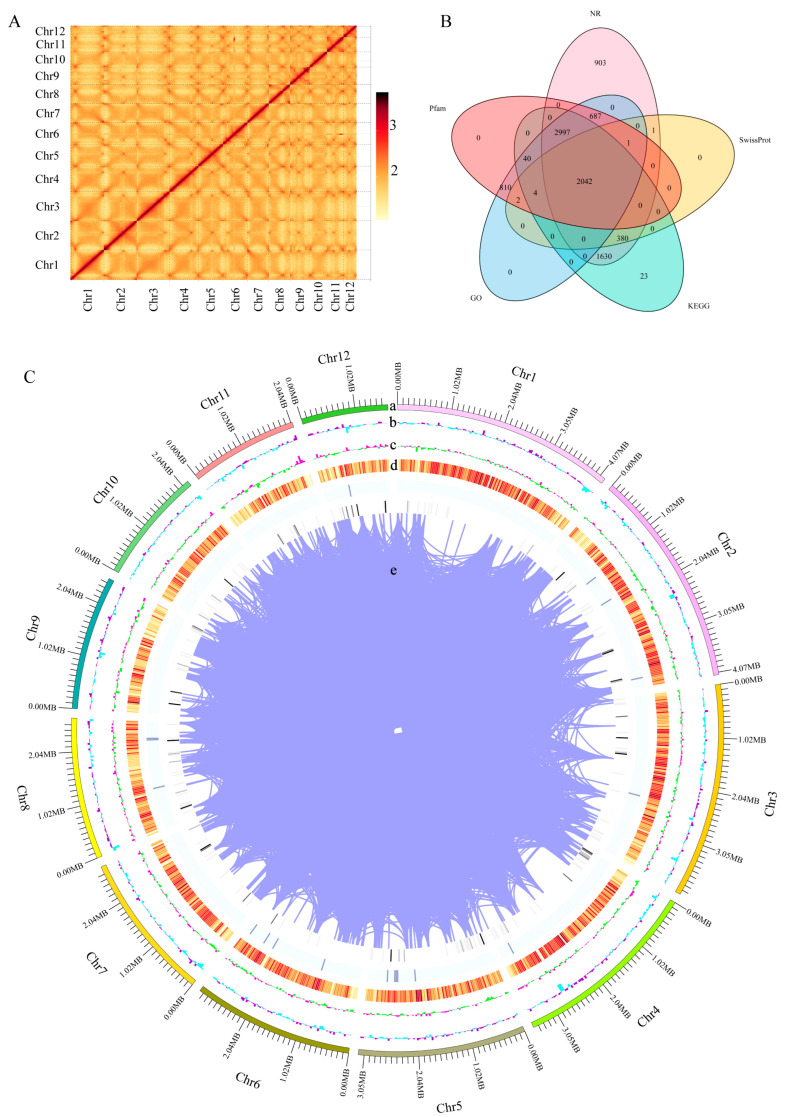
Whole genome assembly and annotation of *F. filiformis*. (**A**) Chromosome-wide Hi-C interaction heat map, (**B**) Annotation of predicted genes in 5 databases (GO, KEGG, Pfam, NR, SwissProt), (**C**) Overview of the *F. filiformis* genome assembly, (a) The 12 assembled chromosomes, (b) GC content per window, (c) GC skew value per window, (d) Gene density, (e) Gene duplication.

This study conducted whole-genome sequencing and HIC-assisted assembly using the mycelium of the monokaryotic strain HNY6m1 of the wild *F. filiformis* HNY6, obtaining high-quality genome assembly results. The genome of HNY6m1 was 36.3 Mb after assembly, containing 24 scaffolds. The scaffolds N50 and N90 were 3.17 Mb and 2.06 Mb, respectively. The lengths of the largest and smallest scaffolds were 4.18 Mb and 3.8 kb, respectively. All genes could be assigned to 12 chromosomes (Table 1). A total of 11,886 genes were annotated in the genome of *F. filiformis*, accounting for 45.05% of the total length of the genome. Among them, the GC content was 52.38%, the average length of the genes was 1378 bp, and the total length of the internal regions of the genes was 20.01 Mb (Table 1). The Hi-C interaction heat map proved that this genome achieved a high-quality assembly at the chromosomal level (Figure 1A).

The functional annotation results in the NR, SwissProt, KEGG, GO, and Pfam databases showed that 8641, 2430, 7116, 6583, and 6583 homologous sequence encoding proteins were identified, respectively, and 2042 proteins of these were annotated in the above five databases (Figure 1B, Table S1). BUSCO analysis showed that 95.9% of fungal core genes were covered on the genome (Figure S1). These data indicate that the assembled high-quality genome of HNY6m1 (Figure 1C) had high completeness and accuracy and can provide an important reference for the study of the gene function and evolution of *F. filiformis*.

### 3.2. Comparative Genomic Analysis of F. filiformis and Other Fungi

**Figure 2:**
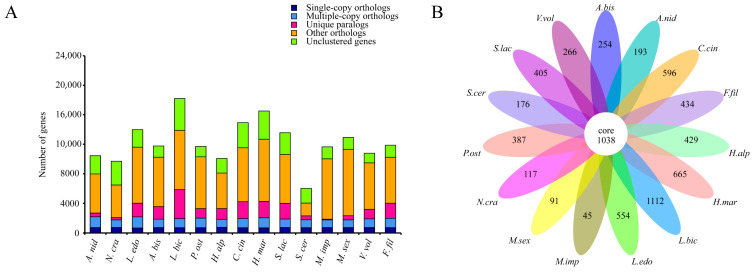
Comparison of genomes between *F. filiformis* and 14 other fungi (9 basidiomycetes and 5 ascomycetes), (**A**) Number of different orthologous gene types in each fungal species, (**B**) Venn diagram of orthologous gene families in multiple species, the data on each region represent unique gene families in this species.

Comparative genomic analyses of the *F. filiformis* genome with 14 other fungal genomes (including nine basidiomycetes and five ascomycetes, Table S2) were conducted. Genes in the genomes were classified according to their copy numbers as single-copy orthologs, multiple-copy orthologs, unique paralogs, other orthologs, and unclustered genes. In the genome of *F. filiformis*, a total of 716 single-copy orthologs, 1271 multiple-copy orthologs, and 2024 unique paralogs were identified (Figure 2A). Additionally, 1038 genes were shared by all 15 fungi, while 434 genes were specific to *F. filiformis* (Figure 2B).

### 3.3. Genome Annotation Analysis

**Figure 3:**
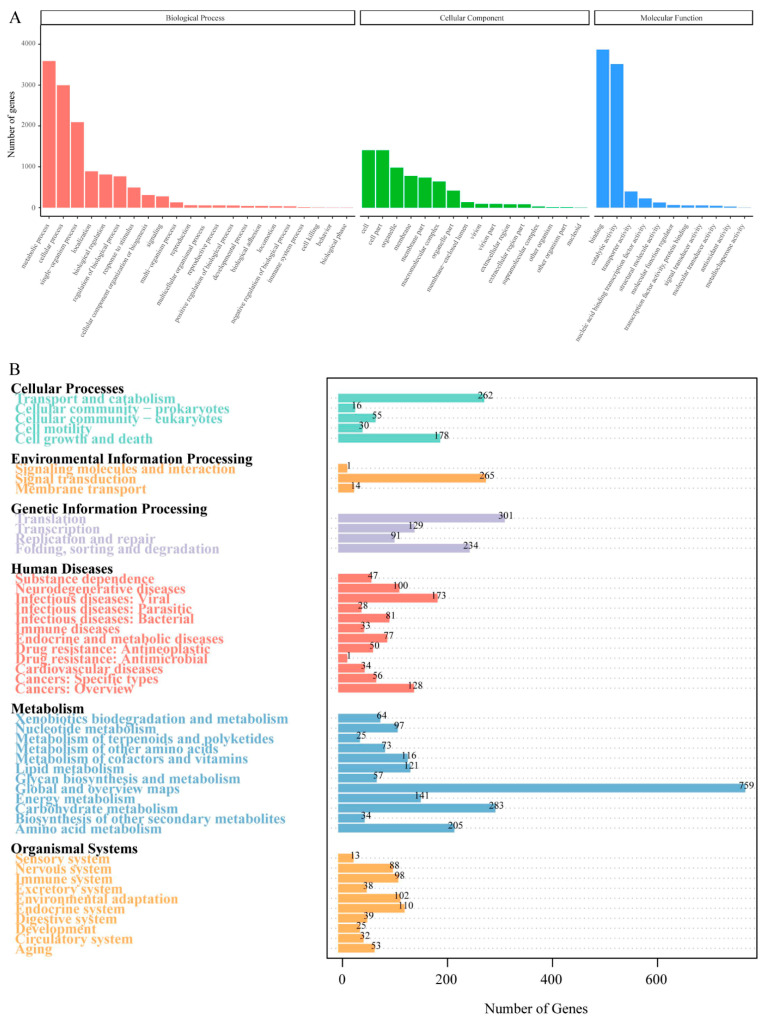
Classification statistics of GO and KEGG annotations of *F. filiformis* genome. (**A**) The GO function annotation of predicted genes in *F. filiformis*, (**B**) The KEGG pathway annotation of predicted genes in *F. filiformis*.

The GO database analysis showed that there were a total of 6583 predicted genes, mainly distributed among the five items: metabolic process, cellular process, single organism in biological processes, and binding and catalytic activity in molecular functions (Figure 3A). The functional classification of the *F. filiformis* genes in the KEGG database is shown in Figure 3B. The 7116 predicted genes of *F. filiformis* were mapped to related pathways such as metabolism, cellular processes, environmental information, and genetic information. The pathways with more genes in the metabolism process include carbohydrate metabolism, amino acid metabolism, and energy metabolism. Genes in the genetic information process are mainly involved in pathways such as transcription, translation, replication and repair, and folding and degradation. The environmental information process mainly includes signal molecules and interactions, signal transduction, and membrane transport pathways. The cellular process mainly includes transport and catabolism as well as cell growth and death pathways, which contain a larger number of genes.

### 3.4. Cold-Responsive Genes and Cold-Adapted Enzymes in the Genome of F. filiformis

**Figure 4:**
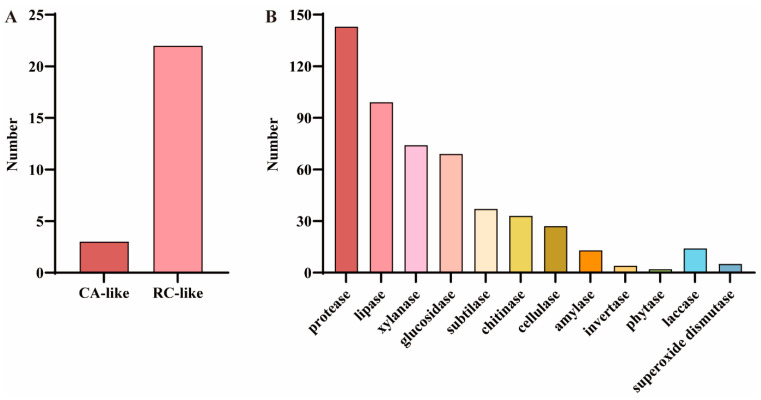
Cold-responsive genes and cold-adapted enzymes in the *F. filiformis* genome. (**A**) GO analysis results of cold-responsive (COR) genes in *F. filiformis*, including cold acclimation (CA) and cold response (RC) categories, (**B**) Analysis results of cold-adapted enzymes in *F. filiformis*.

To identify COR-related genes in *F. filiformis*, the predicted protein sequences associated with cold response in *A. thaliana* were utilized for alignment analysis, drawing on studies in plants. The results showed that a total of 25 predicted protein sequences significantly similar to the COR proteins in *A. thaliana* were identified, among which three were related to CA and 22 were related to RC (Figure 4A and Table S3). In addition, considering the important role of certain enzymes in fungal cold adaptation, a total of 520 cold-adapted enzymes were identified in the genome of *F. filiformis* based on its gene annotation information, among which 501 were hydrolases (including 143 proteases, 99 lipases, 74 xylanases, 69 glucosidases, 37 subtilases, 33 chitinases, 27 cellulases, 13 amylases, four invertases, and two phytases) and 19 oxidoreductases (including 14 laccases and five superoxide dismutases) (Figure 4B and Table S4). In addition, among the cold-adapted enzymes identified in *F. filiformis*, 248 members (accounting for 47.7%) were annotated as CAZymes. These enzymes were mainly distributed among xylanases (74), glucosidases (65), chitinases (33), cellulases (26), amylases (13), and laccases (13) (Table S4).

### 3.5. Analysis of Gene Expression Differences at Low Temperature

**Figure 5:**
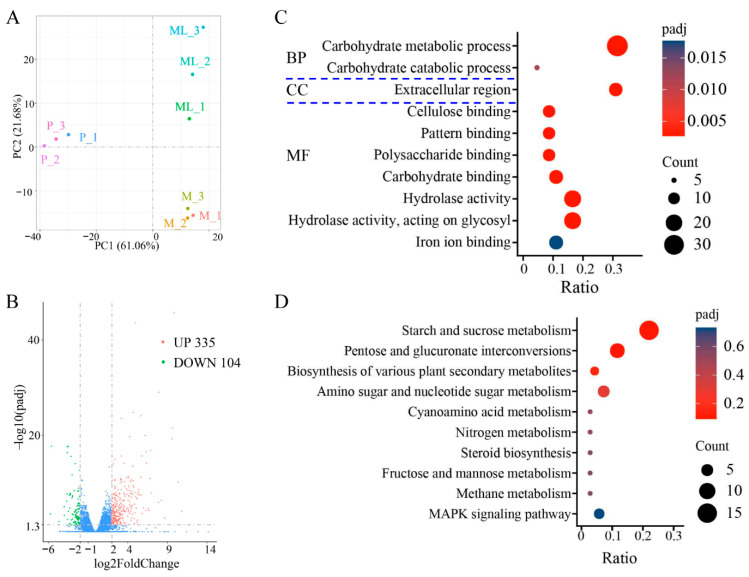
Transcriptome sequencing analysis of *F. filiformis* at different stages. (**A**) Principal component analysis, mycelium cultured at 25 °C (M), mycelium at low temperature conditions (ML), primordium (P), (**B**) Volcano plots displaying the upregulated and downregulated genes between ML and M samples, blue dots indicate no-significantly different genes, (**C**) Gene Ontology (GO) classification of 439 DEGs, (**D**) Encyclopedia of Genes and Genomes (KEGG) enrichment analysis of 439 DEGs.

The optimal temperature for the growth of *F. filiformis* mycelium is 25 °C, but low-temperature stimulation is needed for primordia and fruiting-body formation [8,35]. The results of principal component analysis (PCA) showed that the disparity among different groups (M, ML, and P) was significant, and the samples within the group exhibited good repeatability (Figure 5A). Compared with the mycelium cultured at 25 °C, the mycelium of *F. filiformis* cultured under 10 °C had significant differences in gene expression. When the FC (fold change) reached four times, a total of 439 differentially expressed genes (DEGs) were identified, among which 335 genes were significantly upregulated and 104 genes were significantly downregulated (Figure 5B, Table S6). The differentially expressed genes may play an important role in the cold-adaptive growth process of *F. filiformis* and participate in regulating the physiological and molecular mechanisms necessary for adapting to the low-temperature environment.

The GO enrichment analysis of the above DEGs covers three aspects: cellular composition (CC), molecular function (MF), and biological process (BP). The results showed that the DEGs were predominantly enriched in the following GO categories: carbohydrate metabolic process, carbohydrate catabolic process, extracellular region, cellulose binding, pattern binding, polysaccharide binding, carbohydrate binding, hydrolase activity, hydrolase activity/acting on glycosyl, and iron ion binding (Figure 5C). It is worth noting that the carbohydrate metabolic process dominated in the biological process category. KEGG enrichment analysis showed that the DEGs after cold treatment were mainly enriched in the following metabolic pathways: starch and sucrose metabolism, pentose and glucuronate interconversions, biosynthesis of various plant secondary metabolites, amino sugar and nucleotide sugar metabolism, cyanoamino acid metabolism, nitrogen metabolism, steroid biosynthesis, fructose and mannose metabolism, methane metabolism, and MAPK signaling pathway (Figure 5D). Interestingly, both the GO and KEGG analysis results indicated that the enrichment of differential genes under low-temperature treatment was mainly focused on those related to sugar metabolism.

### 3.6. Analysis of Metabolite Differences at Low Temperature

**Figure 6:**
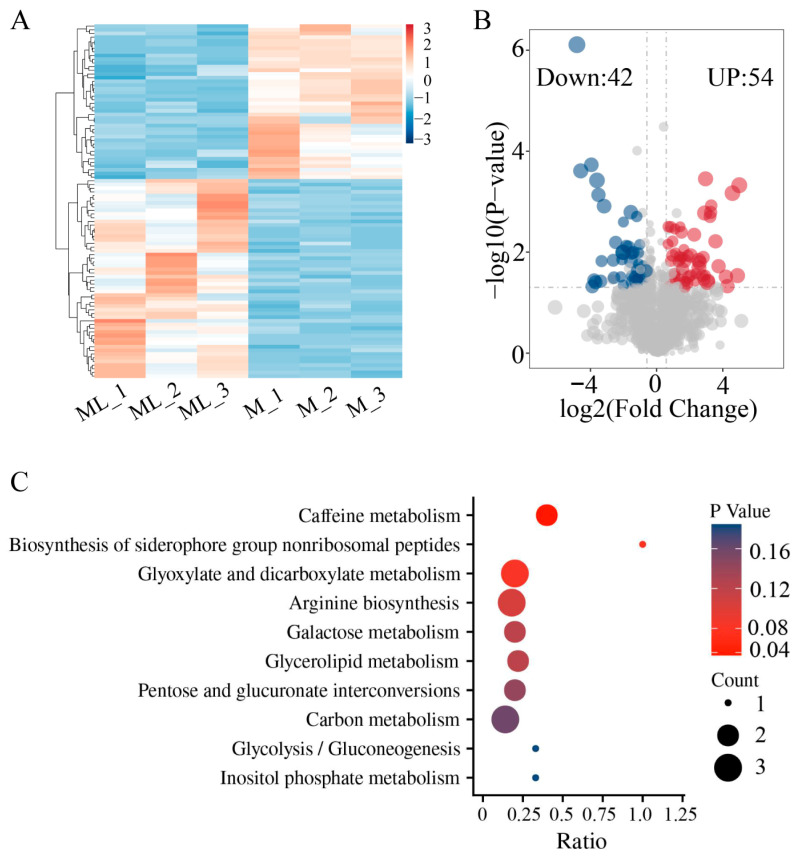
Untargeted metabolomics analysis of ML and M samples of *F. filiformis.* (**A**) Cluster analysis of metabolites, (**B**) Volcano plots displaying the upregulated and downregulated metabolites between ML and M samples, (**C**) KEGG enrichment analysis of significantly different metabolites.

The results of the metabolome analysis indicated that there were significant differences in metabolites between the mycelia samples grown at 25 °C (M) and at 10 °C (ML) (Figure 6A). Compared with mycelia grown at 25 °C, a total of 96 DAMs were identified after low-temperature treatment, among which 42 metabolites were significantly upregulated and 54 metabolites were significantly downregulated (Figure 6B, Table S7). Further functional annotation of the DAMs was conducted through KEGG enrichment analysis, and the results showed that these metabolites were enriched in 10 metabolic pathways, including caffeine metabolism, biosynthesis of siderophore group nonribosomal peptides, glyoxylate and dicarboxylate metabolism, arginine biosynthesis, galactose metabolism, glycerolipid metabolism, pentose and glucuronate interconversions, carbon metabolism, glycolysis/gluconeogenesis, and inositol phosphate metabolism (Figure 6C). Pathways related to sugar metabolism were significantly enriched and accounted for a large proportion, suggesting that sugar metabolism may play a crucial role during the low-temperature adaptation process of *F. filiformis*.

### 3.7. CAZyme Genes in F. filiformis and Its Response to Low Temperature

**Figure 7:**
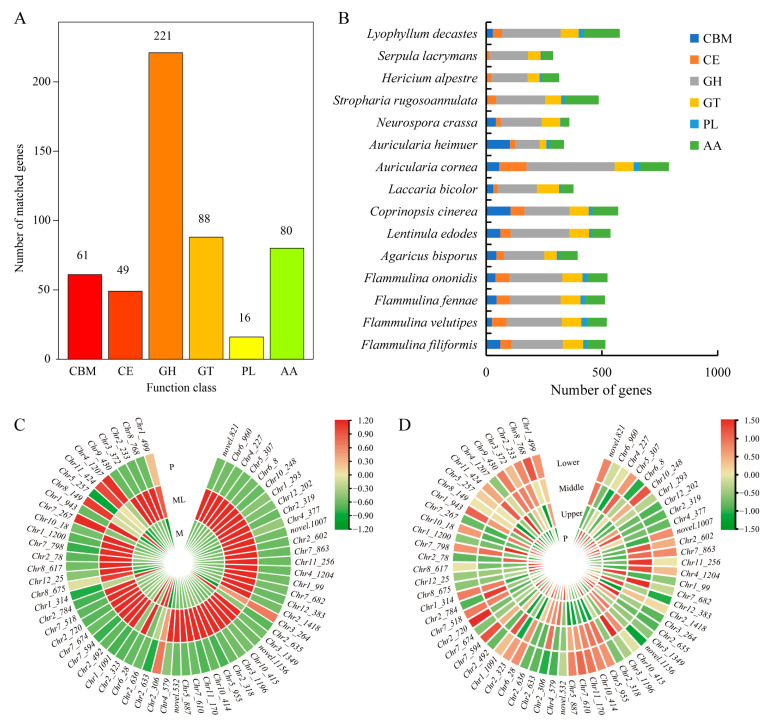
CAZymes in *F. filiformis* and other 14 fungi. (**A**) The distribution of CAZyme families in *F. filiformis*, (**B**) The distribution of CAZymes in all 15 fungi, (**C**) CAZyme genes in DEGs (ML vs. M) and the expression heat map in ML and M samples, (**D**) The expression heat map of CAZyme genes in different parts of *F. filiformis*.

The CAZy database defines six types of enzymes related to carbohydrate metabolism, including glycoside hydrolases (GHs), glycosyltransferases (GTs), carbohydrate esterases (CEs), carbohydrate-binding modules (CBMs), auxiliary activities (AAs), and polysaccharide lyases (PLs). Through the analysis of the *F. filiformis* genome, a total of 515 CAZymes genes were identified (Figure 7A). Among these six types of CAZymes families, glycoside hydrolases (GHs) were the most abundant family (42.9%), which is closely related to the lifestyle of *F. filiformis* and might be because its growth relied on the degradation of lignocellulose. By contrast, polysaccharide lyases (PLs) were the least abundant family (3.1%), which indicates that their role in the growth and metabolism of *F. filiformis* was relatively small. It is worth noting that the number of CAZymes genes and the proportion of each family in the genomes of *F. filiformis* and other mushrooms of the same genus showed similarity (Figure 7B).

At low temperature, a large number of CAZyme genes in the mycelium were activated. Using a fold change (FC) of two as the screening criterion, 64 CAZymes were identified among 1404 DEGs, including 57 GHs, five GTs, one PL, and one CBM (Figure 7C, Table S8). Among them, 24 CAZyme genes were also identified as cold-adapted enzymes in the genome of *F. filiformis*. These included seven glucosidases, four xylanase/glucosidase/cellulase, three cellulase, three xylanase/chitinase, three xylanase/cellulase, one invertase, one xylanase, one xylanase/glucosidase, one glucosidase/cellulase, and one glucosidase/amylase (Table S8). Particularly worth noting was that 58 of these DEGs were significantly upregulated, and the upregulation fold of 28 genes exceeded 10 times. Among these differentially expressed GH family genes, the GH61 family contained 14 genes, and the highest upregulation fold could reach 1418.65 times. Although the expression of these genes significantly increased during the cold-adaptive growth process, after the formation of primordia, the expression levels of these genes gradually decreased, approaching the level of mycelium cultured at 25 °C. Meanwhile, the expression levels of CAZyme genes in various parts of the fruiting body were relatively low (Figure 7D). These results indicate that the CAZyme family of genes plays an important role in the cold-adaptation process of the mycelium of *F. filiformis*.

## 4. Discussion

Currently, research on edible fungi is increasingly transitioning from traditional phenotypic characteristics, nutritional components, biological activity, and cultivation methods toward a molecular-level understanding. This shift aims to analyze internal regulatory mechanisms and improve agronomic traits using molecular biology techniques. In this study, the Illumina NovaSeq platform was utilized for the de novo sequencing and assembly of the wild yellow *F. filiformis* HNY6 monokaryotic strain. The results revealed that the genome size of HNY6 is 36.3 Mb and that a BUSCO completeness of 95.9% was achieved, with a total of 11,886 genes annotated. A search of the NCBI public database reveals that only 10 genomes of *F. filiformis* have been publicly released to date, including four strains (GCA_041682555.1, GCA_022345045.1, GCA_044231765.1, GCA_015342475.1) at the contig level, two strains (GCA_001602335.1, GCA_022345005.1) at the scaffold level, and the other two strains (GCA_041684055.1, GCA_011800155.1, GCA_945909995.2, GCA_000633125.1) at the chromosome level. The genome sizes of the currently available *F. filiformis* genomes range from 34 to 40 Mb. The earliest chromosome-level genome was released in November 2014 (*Flammulina velutipes* KACC42780, GCA_000633125.1) with a total length of 35.6 Mb, including 11 largest scaffolds representing 11 chromosomes [19]. Whole-genome analysis of the HNY6 strain revealed that its genome size and the number of protein-coding genes are consistent with previously published *F. filiformis* genomes. In this study, isolated monokaryotic mycelia were employed for whole-genome sequencing analysis. The dikaryotic form contains two complete and distinct genomes, and the genomic interactions between them may compromise sequencing accuracy and the precision of subsequent analyses. Sequencing the monokaryotic form not only simplifies the analysis but also minimizes redundancy, providing a clearer framework and distinct advantages for investigating genes associated with biological characteristics [36]. Consequently, compared to the dikaryotic form, the sequencing results of the monokaryon exhibit higher accuracy.

Genes associated with metabolic processes were significantly enriched in the genome of *F. filiformis*. This underscores the significance of metabolic activities in its genomic functions and suggests that metabolism-related genes may play a crucial role in its growth and environmental adaptation. The process of fruiting-body formation of *F. filiformis* depends on a low-temperature environment. In this study, a comparative analysis of cold-responsive genes in its genome was conducted, revealing a total of 25 cold-responsive genes, including three CA and 22 RC. In comparison, the genomes of other plants, such as *Malus baccata*, *Malus domestica*, and *Solanum commersonii*, contain 3978, 5089, and 2860 cold-responsive genes, respectively [32,37]. These differences may be attributed to the smaller genome size of *F. filiformis*. The genome of the *F. filiformis* HNY6 strain is 36.3 Mb, whereas those of *M. baccata* (665.8 Mb), *M. domestica* (603.9 Mb), and *S. commersonii* (830 Mb) are significantly larger [32,37,38]. These differences in genome size may lead to variations in the number and diversity of cold-responsive genes. The cold-adaptation ability of *F. filiformis* may rely on relatively simple mechanisms, such as metabolic pathways like osmotic pressure adjustment or cellular protection mechanisms like activating the antioxidant system and improving protein stability, to enhance its cold resistance. The results of cold-adapted enzymes further support this hypothesis. Saprophytic fungi actively decompose organic matter by secreting various hydrolases, thereby participating in the cycling of carbon and nutrients in cold environments [39]. Additionally, oxidoreductases can eliminate reactive oxygen species induced by low temperature, maintaining the redox balance in cells [40]. In this study, 520 cold-adaptation-related enzymes were identified in the genome of *F. filiformis*, including 248 CAZymes related to matrix degradation, which may play specific biological functions during its unique growth, development, and morphogenesis at low temperatures.

Low-temperature stimulation is a crucial environmental factor influencing the transformation of *F. filiformis* mycelium into primordia, thereby impacting the yield and quality of fruiting bodies. This study employed transcriptome and metabolome analyses to examine *F. filiformis* mycelium under conditions of 25 °C and 10 °C. The results of DEG enrichment indicated that cold stimulation predominantly affected metabolic processes. Concurrently, DAM enrichment revealed significant enrichment in sugar metabolism-related pathways, suggesting that *F. filiformis* enhances energy metabolism during low-temperature adaptation to supply the necessary energy and metabolic substrates for the cold-adaptation process. Previous research reports demonstrated that in a proteomic analysis conducted by comparing the mycelia of *F. filiformis* at room temperature (25 °C) and low temperature (12–15 °C), 13 upregulated proteins and 100 downregulated proteins were identified following cold treatment (FC = 1.5) [4]. This finding contrasts with the transcriptome analysis in the present study, where a screening threshold of FC = 2 revealed that 839 genes were upregulated and 565 genes were downregulated (Table S5), while a screening threshold of FC = 4 revealed that 335 genes were upregulated and 104 genes were downregulated. Furthermore, the metabolomic analysis in this work revealed that 42 metabolites were upregulated while 54 metabolites were downregulated (FC = 1.5), aligning with the trends observed in the proteomic data. We speculate that *F. filiformis* initiates extensive transcriptional activity in response to low-temperature stress, leading to notable alterations at the transcriptional level. However, low temperature may directly suppress protein synthesis or activity, preventing these transcripts from translating into proteins, thereby constraining the extent of metabolite alterations. In addition, different strains or varieties of *F. filiformis*, culture conditions, and sampling times might cause variations in the results of multi-omics analysis. The transcriptomic analysis of *F. filiformis* mycelium under room temperature and primordia under low temperature revealed that two-component signal pathways, calcium signal pathways, mitogen-activated protein kinase pathways, molecular chaperones, and the cell wall and membrane systems play crucial regulatory roles during the formation process of the primordia [23]. By contrast, this study primarily identified significant changes in sugar metabolism-related genes during the low-temperature adaptation process, suggesting that the cold stimulation adaptation and cold-induced fruiting processes may rely on different molecular regulatory mechanisms. During the cold-adaptation stage of *F. filiformis* mycelium, energy acquisition and carbon source metabolic pathways might be crucial links in responding to low temperature, and the growth and metabolic balance could be maintained by regulating genes related to sugar metabolism. During the cold-induced fruiting stage, complex signal pathways and molecular chaperone systems might predominate in the regulation of cell proliferation, differentiation, and fruiting processes at low temperature.

Our analysis highlights the significant role of CAZymes in the cold-adaptation process of *F. filiformis*. CAZymes, essential for carbohydrate biosynthesis and metabolism [41], are vital for cell life and enable basidiomycetes to hydrolyze lignocellulosic biomass effectively [42]. These CAZymes are implicated in the hydrolysis of polysaccharides within plant cell walls, and the degraded substrates can act as nutrients to sustain the growth and development of fungi [43,44]. In the *F. filiformis* genome, the GH family gene content accounts for nearly half of the CAZymes, which indicates that *F. filiformis* may rely on the ability of lignin degradation as a key adaptation mechanism for its survival and growth. Notably, GH family genes constitute the largest proportion of CAZyme families in other species of basidiomycete, often nearing or exceeding 50% [14,15,17,19,45], highlighting their efficacy in decomposing plant materials like lignocellulose and lignin. Previous work reveals that blue light enhances the enzymatic activity of CAZymes, aiding in fruiting-body differentiation and development [44]. CAZymes are involved not only in substrate decomposition but also in the formation of sclerotia in *Wolfiporia cocos* and *Pleurotus tuberregium* [36,46]. Under low-temperature culture conditions, the activities of lignocellulose-degrading enzymes such as laccase, xylanase, and CMCase in *P. ostreatus* were significantly enhanced [47]. These enzymes can effectively break down lignin and cellulose in the culture medium, releasing more nutrients for mycelium growth. The transcriptome analysis conducted in this study revealed that exposure to low temperature significantly upregulated the expression of genes related to CAZymes, potentially enhancing nutrient utilization efficiency in the medium through increased enzyme activity. Notably, the GH61 family exhibited upregulation in 14 genes, constituting 22% of the differentially expressed CAZymes genes. Enzymes of the GH61 family, which are prevalent in fungi, possess limited cellulose degradation activity. However, when functioning synergistically with other cellulases, they can substantially enhance the hydrolysis efficiency of cellulose. Consequently, GH61 is classified as a lytic polysaccharide monooxygenase (LPMO) and further categorized within the auxiliary activity family (AA) [48,49]. It is speculated that GH61 may, during the process of low-temperature adaptation, regulate carbon source utilization by augmenting cellulose degradation capacity, thereby providing additional energy and metabolites to facilitate *F. filiformis*’s adaptation to environmental changes. In low-temperature environments, the mycelium of *F. filiformis* may regulate the expression of CAZymes to enhance carbon source utilization, maintain extracellular enzyme activity, and ensure mycelial growth stability. Therefore, these results will provide a valuable reference for the study of the molecular mechanism of the low-temperature adaptation of *F. filiformis* and will also provide clues for the traditional breeding and molecular breeding of new varieties with high yield and high biological efficiency.

## 5. Conclusions

This study conducted de novo genome sequencing of the *F. filiformis* HNY6 monokaryotic strain and predicted 11,886 genes based on the genomic data. These genes were annotated using various protein databases, and subsequent GO and KEGG enrichment analysis results showed that “metabolism”-related genes accounted for a high proportion, indicating that *F. filiformis*, as a saprophytic organism, adapts to its living environment through strong metabolic activity to obtain external nutrients. Under low-temperature conditions, *F. filiformis* showed obvious changes in sugar metabolism activity, which suggests that sugar metabolism may support its physiological adaptation process by providing energy under low-temperature stress. In particular, the high expression of CAZyme genes was only observed in the low-temperature mycelium, further indicating the crucial role of this type of gene in low-temperature cold adaptation. Overall, the results of this study provide a new perspective and theoretical basis for further revealing the adaptation mechanism of *F. filiformis* mycelium in regulating low-temperature stress during the growth and development process.

## Figures and Tables

**Table 1:** Genome assembly features of monokaryotic *Flammulina filiformis*.

Characteristics	Number/Length
Scaffold characteristics	
Total number	24
Total length	36.3 Mb
N50 length	3.17 Mb
N90 length	2.06 Mb
Max length	4.18 Mb
Min length	3.8 kb
Sequence GC	49.50%
Genome characteristics	
Genome assembly size	36.3 Mb
Chromosome number	12
Gene number	11,886
Gene length	16.4 Mb (45.05% of the genome)
GC content	52.38%
Gene average length	1378 bp
Gene internal length	20.01 Mb

## Data Availability

This Whole Genome project has been deposited at GenBank under the accession GCA_041684055.1.

## Supplementary Materials

The following supporting information can be downloaded at: https://www.mdpi.com/article/10.3390/jof11030229/s1, Table S1: Annotated information of genes in 5 databases; Table S2: List of fungal genomic data sources used in this study; Table S3: Identified cold-responsive (COR) gene annotation information; Table S4: Identified cold-adapted-enzyme gene information; Table S5: DEGs between ML and M samples (FC = 2); Table S6: DEGs between ML and M samples (FC = 4). Table S7: DAMs between ML and M samples (FC = 1.5); Table S8: CAZyme genes in DEGs. Figure S1. BUSCO assessment of the protein annotation completeness in *Flammulina filiformis*. The completeness of gene prediction was 95.9%.
