# Supplementary material for: Whole-Genome Sequencing of Flammulina filiformis and Multi-Omics Analysis in Response to Low Temperature
Source: J Fungi (Basel). 2025 Mar 17;11(3):229. doi: 10.3390/jof11030229 (PMC11942922; doi:10.3390/jof11030229)
Supplement: Supplementary file 1 [file jof-11-00229-s001.zip › Supplemental Figure.pdf]

Supplementary figure

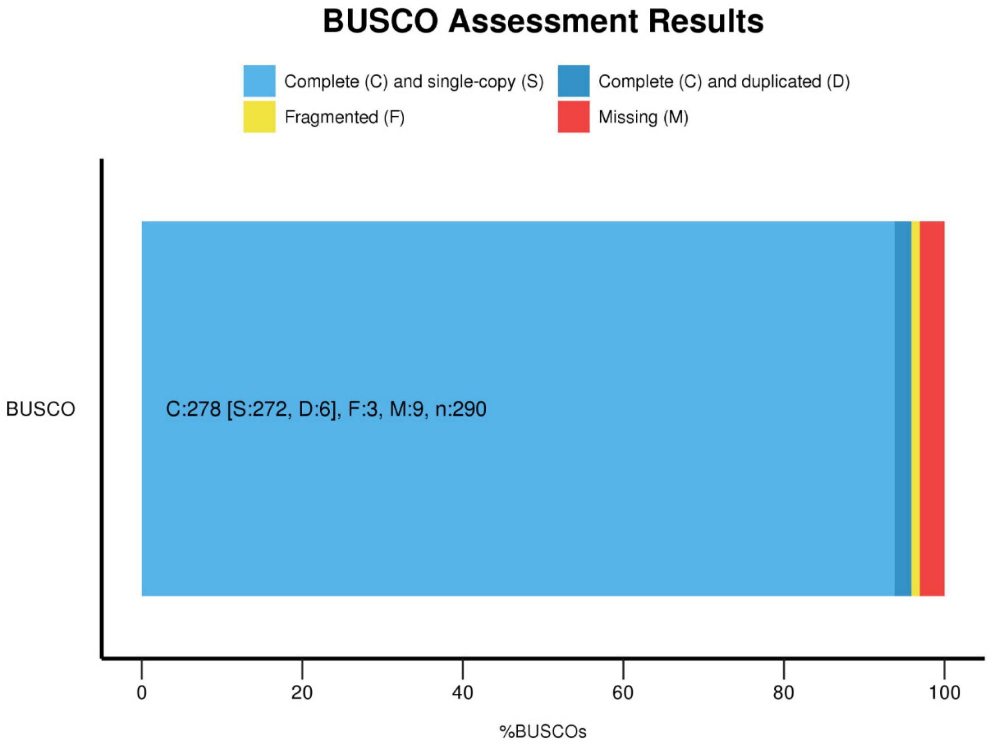

Figure S1. BUSCO assessment of the protein annotation completeness in *Flammulina filiformis*. The completeness of gene prediction was 95.9%.
